# Production, characterization and performance of green geopolymer modified with industrial by-products

**DOI:** 10.1038/s41598-024-55494-8

**Published:** 2024-03-01

**Authors:** Ramadan Abbas, M. A. Abdelzaher, Nabila Shehata, M. A. Tantawy

**Affiliations:** 1 Environmental Science and Industrial Development Department, Faculty of Postgraduate Studies for Advanced Sciences, Beni-Suef University, Beni-Suef, 62511 Egypt; 2 Chemistry Department, Faculty of Science, Minia University, Minia, Egypt

**Keywords:** Industrial by-products, Metakaolin geopolymer, Physico-mechanical characteristics and microstructure, Composites, Mechanical properties

## Abstract

Industrial by-products; have received a lot of attention as a possible precursor for cement and/or concrete production for a more environmentally and economically sound use of raw materials and energy sources. Geopolymer is a potentially useful porous material for OPC binder applications. The use of industrial wastes to produce a greener geopolymer is one area of fascinating research. In this work, geopolymer pastes were developed using alkali liquid as an activator and metakaolin (MK), alumina powder (AP), silica fume (SF), and cement kin dust (CKD) as industrial by-products. Several geopolymer samples have been developed. Research has been carried out on its processing and related physical and mechanical properties through deep microstructure investigation. The samples were cured in water by immersion with relative humidity (95 ± 5%), and at room temperature (~ 19–23 °C) prior to being tested for its workability and durability. The effect of the different composition of precursors on water absorption, density, porosity, and the compressive strength of the prepared geopolymers have been investigated. The results showed that the compressive strength of geopolymers at 28 days of curing is directly proportional to the ratio of the alkali liquid. Ultimately, the best geopolymer paste mixture (GPD1 and GPD2), was confirmed to contain (15% of CKD + 85% MK and Alumina solution (55 wt%)) and (25% of CKD + 75% MK + Alumina solution (55 wt%)) respectively, with 73% desirability for maximum water absorption (~ 44%) and compressive strength (4.9 MPa).

## Introduction

**Figure 1:**
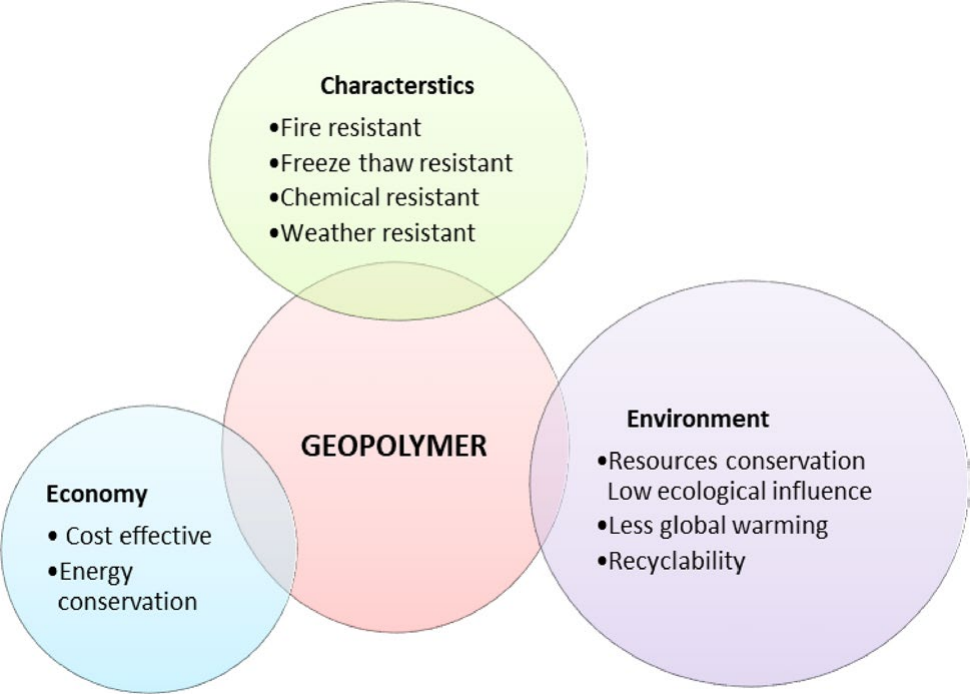
The circular economy of geopolymer composite materials.

Cement industry causes high carbon emissions (about 7% of global CO_2_ emissions)^1^, and has high energy costs^2^. Urban expansion causes an increase in the demand for cement, which is followed by an increase in carbon di-oxide emissions that cause serious environmental pollution and climate change problems. One of the alternatives that contribute to solving these problems is the development and production of activated alkali cementor geopolymers, which significantly contributes to reducing carbon dioxide emissions. This confirms the multiple environmental benefits related to the application of these sustainable binders^3^, less energy consumption and less environmental pollution. Figure 1 shows the contribution of the geopolymer composite materials in the circular economy.

Geopolymer cement (GPC) is a competitive alternative to OPC cement in a number of applications. GPC is often used in infrastructure projects, such as bridges and roads, because of its superior durability and mechanical properties^4^. GPC is also being used in a variety of other applications, such as building construction, precast cement products, and fire protection materials. They offer a number of advantages over OPC, including reduced CO_2_ emissions, increased durability, more resistant to fire, corrosion, and acid rain than OPC cement^5,6^. Geopolymers can be produced using a variety of byproducts or recycled materials, such as metakaolin, cement kiln dust and alumina^7–12^. Moreover, the use of alumina, cement kiln dust and silica in the formation of geopolymers can achieve environmental sustainability and reduce industrial waste^13,14^. This research discusses the study of the effect of physical and chemical properties on metakaolin geopolymer when replacing part of the metakaolin with other materials such as alumina, silica fume, and cement kiln dust. This highlights the importance of continuous research in this field and the development of new technologies for using geopolymers and their derivatives in the construction industry, which can be classified as follows:

Kaoline is clay made from kaolinite Al_2_O_3_.2SiO_2._2H_2_O that contains high alumina content ranged between 25 to 40%. The structural unit of kaolinite consists of single tetrahedral silica and octahedral alumina sheets bonded together by hydrogen bonding^15^. Metakaolin is a pozzolanic material that is produced by calcining kaolin clay at temperatures of 600–800 degrees Celsius. Metakaolin geopolymers are known for their high compressive strength and durability^16–19^.

Silica fume is a by-product of the silicon and ferrosilicon industries. It is a very fine powder that consists of amorphous silica. Silica fume is often used in geopolymer formulations to improve the compressive strength and durability of the geopolymer^20–23^. Despite the fact that silica fume has demonstrated several uses as an amorphous supplementary cementing material, few studies focused on using it as a source of reactive and amorphous silica for activators raw material in geopolymer technology^24^. Because of this, the recently published studies sought to determine if silica fume-based sodium silicate is effective in the manufacturing of geopolymers. To do this, silica fume was dissolved in a concentrated 10 M sodium hydroxide solution to create a Sodium Silicate Alternative (SSA)^25–27^. Alkaline Activator (AA) was created by mixing a 1:1 volume ratio of SSA with a 10 M sodium hydroxide solution to create the geopolymer pastes. For MK and GGBS pastes, the AA/PM weight ratios were 1.5 and 0.58, respectively. The outcomes were contrasted with comparable geopolymer binder compositions prepared using sodium silicate (SSC) solution from a commercial source at an identical SiO_2_/Na_2_O molar ratio of 2%. The tests for fresh paste setting times, density, water absorption, compressive strength of hardened pastes after 3, 7, and 28 days, and tensile strength after 28 days of moist curing were the basis for the next analysis, which compared engineering performance^28^. Furthermore, thermogravimetric and deferential thermo-gravimetric (TG/DTG) behavior of hardened pastes at 28 days of moist curing, up to 1000 °C, were compared in order to facilitate the construction of hypotheses for the reaction products.

Cement kiln dust (CKD) is a waste product generated by the cement manufacturing process. It is primarily composed of alumina (Al_2_O_3_) and silica (SiO_2_), with small amounts of other oxides such as iron oxide (Fe_2_O_3_) and magnesium oxide (MgO). The importance of CKD in geopolymer is attributed to the abundance and low cost where it is available in large quantities and at relatively low cost, making it an economical source for geopolymer production. Moreover, the CKD possess suitable physical and chemical properties for geopolymer production, as it contains a high proportion of alumina and silica, which make it a strong binder. In addition, the CKD is characteristic with its reactivity with polymers; it reacts with polymers to form a solid with good mechanical properties, as alumina and silica bind the polymers together^29,30^.

Alumina by-products are generated from a variety of industries, including the aluminum, petroleum, and bauxite industries. Alumina by-products can be used in geopolymer formulations to improve the compressive strength, durability, and fire resistance of the geopolymer^31–33^.

The main objective of the current study is to evaluate the use of such industrial by-byproducts as silica fume, CKD, alumina powder, and metakaolin which are resource/mixing materials to prepare green geopolymer pastes for the OPC binder application. The compositions of precursors on water absorption, apparent porosity, and compressive strength of the prepared geopolymer have been investigated. Despite studies on porous geopolymers for evaporative cooling applications, investigation on the development of green geopolymers requires extra research work. This research developed new green geopolymer binders using different industrial wastes as a greener alternative to OPC binders. Furthermore, capillary height characteristics of selected samples were determined to confirm their suitability as green geopolymer materials for OPC paste binder application.

## Materials and methods

### Materials

Kaolin (5.0 kg) was obtained from the Ceramic Atelier of the Faculty of Fine Arts, (Minia, Egypt). Kaolin (Al_2_O_3_.Fe_2_O_3_. 2H_2_O) was in the form of ultra-fine, reddish-brown powder. The kaolin was fired at 850 °C for 6 h twice, followed by slow cooling at the oven. The resulting metakaolin powder was milled and screened to disperse the agglomerates resulting from adhesion of the kaolin granules during firing, to maintain the high degree of fineness. Sodium silicate was purchased from LUBA Co. (Egypt), and aluminum oxide, was obtained from the Ceramic Atelier of the Faculty of Fine Arts, (Minia, Egypt) . The technical specification of starting materials (as received), are reported in Table 1. The ratio of silica to sodium oxide is 1:1. Sodium silicate solution was prepared by dissolving 1000 g of sodium silicate in a liter of distilled water and set the solution for 24 h before using it as an alkaline activator for geopolymer preparation. Table 2, report the chemical composition of industrial by-products used for the fabrication process.

**Table 1:** Technical specification of sodium metasilicate and aluminum oxide (as received).

Specification	Sodium metasilicate (Na_2_SiO_3_)	Aluminum oxide (Al_2_O_3_)
Appearance	White, uniform, beaded powder	White, powder
Molecular weight ,g	122.69	233.00
Bulk density , g/cm^3^	0.85–0.95	3.98 ± 0.02
Solubility	Soluble in water	Insoluble in water
Degree of polymerization	75–95%	1000 ± 50
Melting point °C	71	2,072
Particular size (14–40 mesh) by wt %	90	66

**Table 2:** The chemical composition of industrial by-products.

Elements / Percent %	SiO2%	Al2O3%	CaO%	Fe2O3%	MgO%	SO3%	Na2O%	K2O%	Cl-%
CKD	14.66	4.17	46.24	2.38	0.97	1.91	5.28	4.37	9.81
SF	93.66	0.92	0.55	0.98	0.33	0.10	0.20	0.31	0.09
AP	1.11	25.97	34.96	5.66	Nil	0.21	0.01	0.02	0.26

### Preparation of geopolymer composites

**Figure 2:**
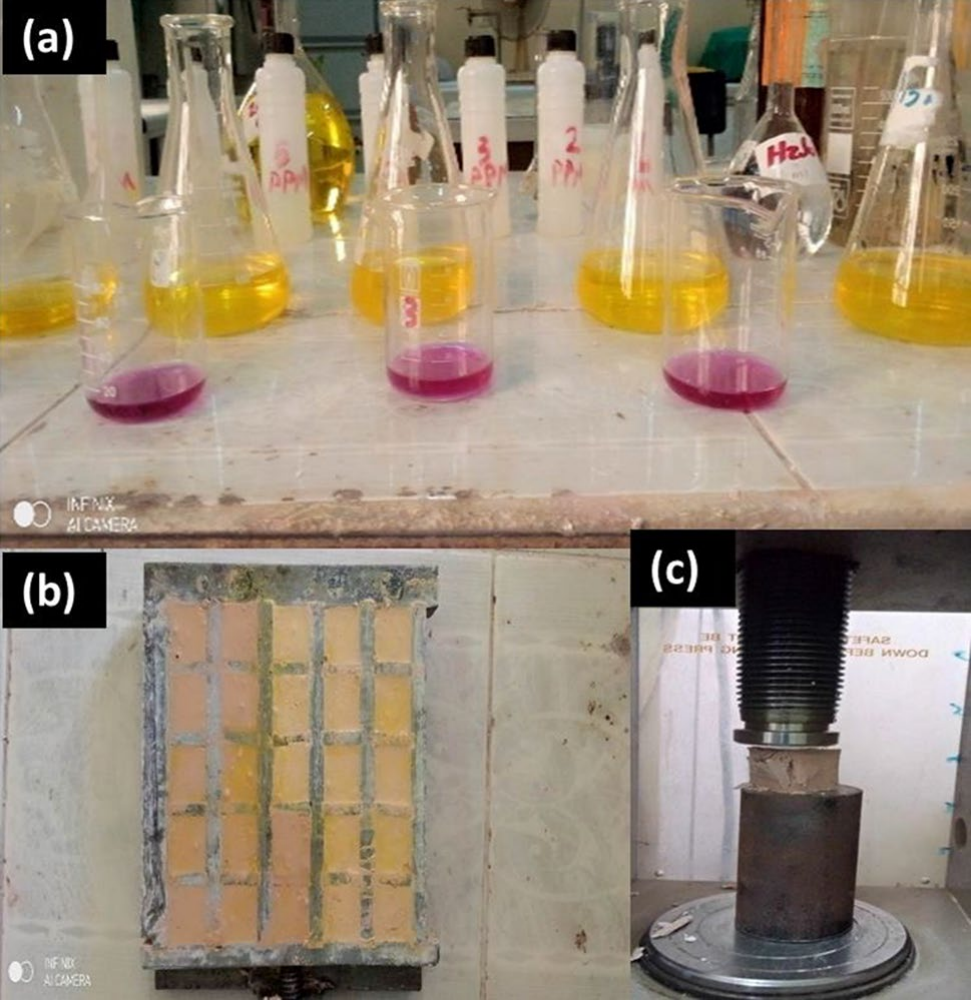
Visual inspection of geopolymer (**a**) Preparation, (**b**) Casting and (**c**) Testing.

Metakaolin geopolymer (MKP); paste composites were prepared by adding 55.0 mL sodium silicate solution to 125 g metakaolin in a stainless steel container. The mixture was mixed and stirred well by hand using a glass foot until dough of good consistency was formed. The dough was poured into 2.0 × 2.0 × 2.0 cm^3^ specimen. The specimen was shaken well after casting to remove air bubbles and better homogeneity. Specimens were kept in a relative humidity cabinet about 95 ± 5%. The geopolymer pastes cubes were de-casted 24 h later. Each individual result represents the average of the three hardened geopolymer paste (repeated tests performed), to avoid variation. A statistical error analysis is calculated for each curing age and each physicochemical analysis, which is shown in graphs 3, 4, 5 and 6. Four mixtures of geopolymers were prepared as shown in Table 3. Figure 2 shows Fig. 2. Visual inspection of geopolymer a) Preparation, b) Casting and c) Testing.

**Table 3:** The proposed mix composition of metakaolin geopolymer composities.

Geopolymer Composition	Alkaline Solution by weight	Metakaolin (MK)	Alumina by weight	Silica fume by weight	Cement kiln dust by weight	Si/Al ratio
GPM	55.0	100.00	–	–	–	–
GPA	55.0	75.00	25.0	–	–	1.21
GPS	55.0	75.00	–	25.00	–	2.13
GPD1	55.0	85.50	–	–	15.00	1.55
GPD2	55.0	75.00	–	–	25.00	1.84
GPD3	55.0	65.50	–	–	35.00	1.99
GPD4	55.0	50.00	–	–	50.00	2.25

### Testing and analysis of prepared geopolymer composites

At ages of 3, 7, and 28 days of casting, the following physical properties were measured: mechanical strength, weight loss, shrinkage percent, bulk density, total porosity, and water absorption percentage. The weight loss and shrinkage percent were measured by calculate the average weights and lengths of three specimens for each geopolymer composite, then the specimen were dried in a desiccator rate of 100 °C for 2 h, and the specimens were cooled, and their weights and lengths were re-measured after reaching drying. Drying weight loss and drying shrinkage percentage were then calculated, by dividing the difference in weight or character length by the original weight or length and multiplying by 100%. Mechanical tolerances were measured using a manual hydraulic specimen, by using 5.00 tons’ load, with a high loading rate of 20.00 kg min^−1^^34–36^. Bulk density and total porosity of an average of three specimens of fracturing products for each geopolymer composite were measured using Archimedes' buoyancy law^37^. Finally, the sample total porosity is given as follow:

1 $$Porosity = \, \left( {\frac{{m_{3} - m_{1} }}{{m_{3} - m_{2} }}} \right) \times 100$$

According to the records, the pore sizes (macro-pores large than 3300 nm, micro-pores in 0–15 nm, while meso-pores in range between 15 and 3300 nm) were investigated^37^. The dry weight of the pieces was measured, then these pieces were immersed in diesel liquid (benzene); for 4 h until the diesel liquid replaces the pores of the samples, then the samples saturated with diesel liquid were weighed suspended in the liquid, and finally the samples were dried with a towel soaked in diesel liquid to remove excess liquid suspended on the surfaces. Then the samples saturated with diesel liquid were weighed. From the three weights, the total density and total porosity were calculated, as shown elsewhere^38^. The percentage of water absorption was measured for an average of three pieces of fracturing products of each geopolymer sample by measuring the dry weight of the pieces, then the pieces were immersed in distilled water for 4 h until the water replaces the pores of the samples, then the samples saturated with water were weighed. The percentage of water absorption was calculated by dividing the difference in weight by the original weight and multiplying by 100%. The remaining samples were kept in sealed containers to protect them from carbonation for instrumentation analyzes were made^39^.

### Characterizations

Oxides content of the silicon and aluminium species present in the selected samples, GPM, GPM, GPS and GPD were investigated using XRF analysis (fluorescence spectrometer), model Philips PW1606. After 28 days’ hydration, the samples were prepared as finely ground powder to reveal the accurate information on the phase identification of the sample, and acquired by a scanning rate of 10 per min from 0 to 600 at a scanning angle (2θ) using XRD instrument, Model Philips PW 1370, with a generated radiation of Cu-kα in 40 mA and 45 kV at room temperature. TGA/DTGA was analyzed by a Shimadzu Corporation thermal analyzer (DTG-60H) at a 10 °C/min heating rate up to 1000 °C, under air atmosphere. FTIR analysis was performed with a Perkin Elmer FTIR apparatus (Spectrum X) in the range 400–4000 cm^−1^. SEM analysis was performed by an electron microscope (Jeol-Dsm 5400 LG), integrated with EDXA namely “an energy dissipation X-ray analyzer”.

## Results and discussions

### Physico-mechanical features

#### Mechanical compressive strength (MCS)

**Figure 3:**
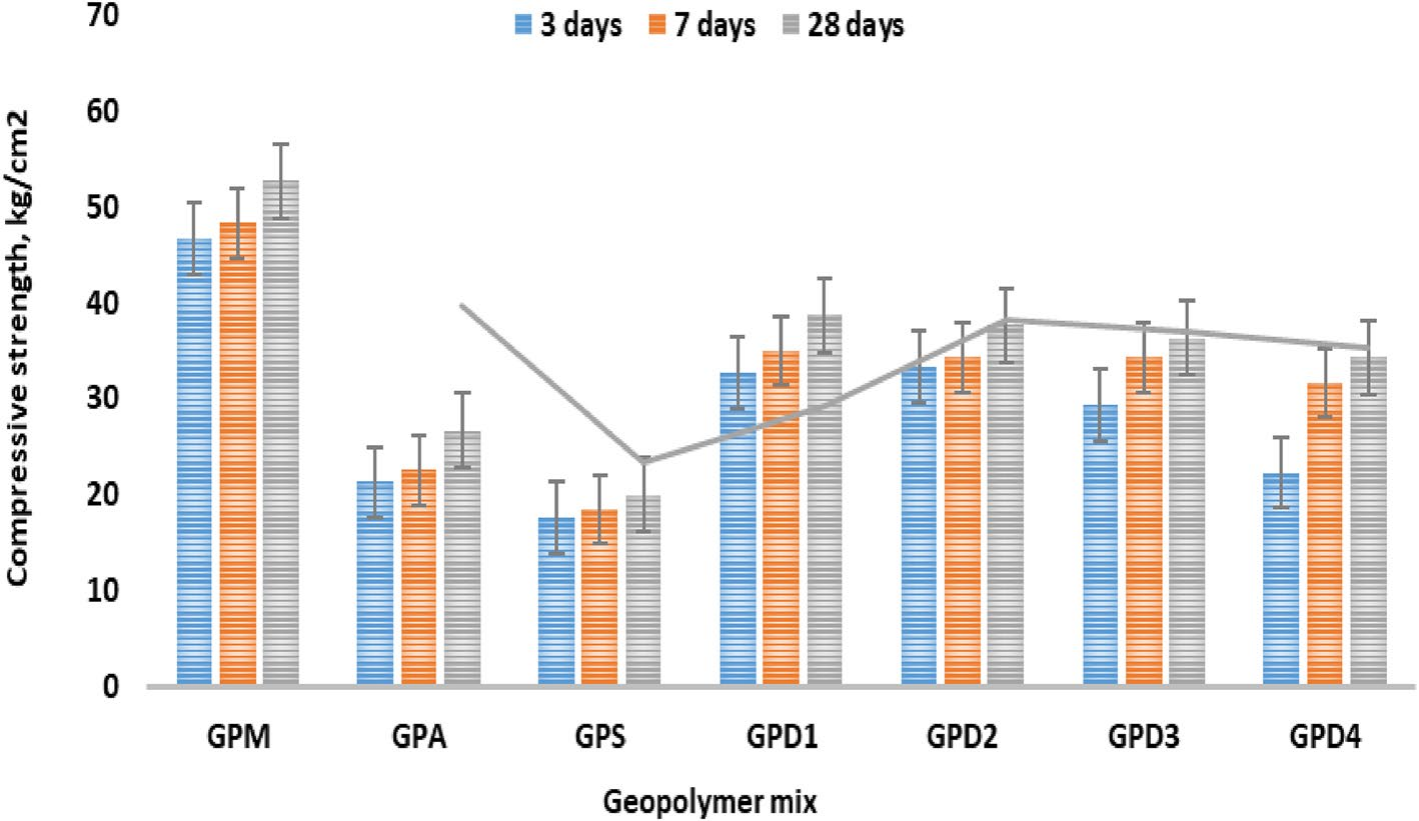
Compressive strength variation of geopolymer pastes hydrated for 3, 27 and 28 days.

Figure 3, illustrates the impact of AP, CKD and SF addition on the MCS of the metakaolin geopolymer composite throughout a late hydration period as a function of hydration ages. According to the data recorded, as more C–A–H and C–S–H (solidification phases) continue to solidify and are completely deposited inside the open pores, the MCS of all geopolymer pastes increases as setting times lengthen resulting in an improvement in the compactness of the specimen microstructure. The MCS of the metakaolin geopolymer composite were improved by the addition of CKD (15.0%); this attributed to the excess CaO, acts as active dynamic filler, improving the microstructure of the blends, moreover; the free alkali content plays an important role during hydration. On contrast; SF (25%); has poor effect during the hydration process, despite of, presence of free silica. The gels C–S–H, C–A–H, and C–A–S–H that comprise the compacted microstructure of the hardened metakaolin geopolymer matrices are developed by the interaction of hydration between CKD and free Alumina and calcium hydroxide^40–42^. However, adding CKD is recommended to a particular amount (35.0%) because anything higher than this could damage the composite's microstructure and have an adverse effect on the matrix, as shown in Fig. 3. The outcomes also showed that the control sample's GPM; was higher compared than proposed metakaolin geopolymer composites incorporated with Al; from both CKD and SF. The metakaolin geopolymer is in the manner, GPM˃GPD1 ˃GPD2 ˃GPD3 ˃GPD4 ˃GPA˃GPS.

#### Bulk density (BD)

**Figure 4:**
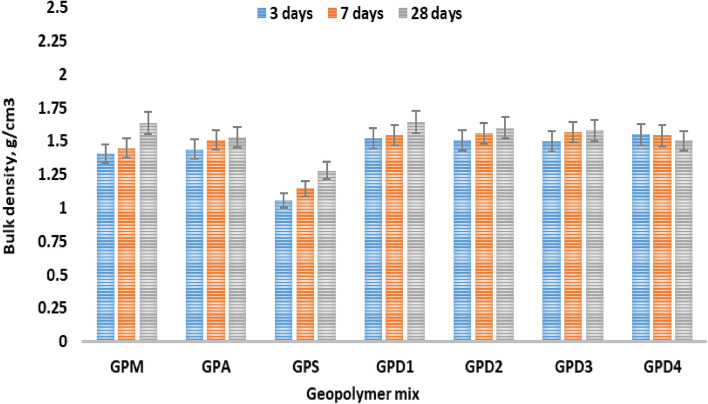
Bulk density of geopolymer variation hydrated for 3, 27 and 28 days.

In Fig. 4, it is depicted how fillers decrease and/or increase the overall bulk density of proposed geopolymers pasts individually. It can be seen that BD increases with CKD addition, which may be attributed to the alkali and calcium oxide content promotes the hydration of excess hydrates, moreover; the high surface area of CKD files the open pores of geopolymers pastes^43,44^. Both SF and AP have limited role during hydration process, but high surface area plays an active role in filling the open pores. It can be assumed that CKD have double activity than SF and AP, as geopolymers haven’t any addition hydration products during hydration process for 3, 7 and 28 days^45^. Geopolymers pastes have the follows order regarding their BD; GPM > GPD1 > GPD2 > GPD3 > GPD4 > GPA > GPS.

Figure 4, also shows that the low rate of BD increases with the progress in hydration ages, which may be attributed to the less activity of the fillers and only effective surface area promotes BD, during late hydration. CKD shows good role due to the high surface area and alkali content^46^. The porosity values of the geopolymers composite e.g.; GPM, and the CPD mixes are high dense than the other pastes^47,48^. The geopolymers pastes have the follows order regarding their BD; GPM > GPD > GPA > GPS.

#### Total porosity (TP)

**Figure 5:**
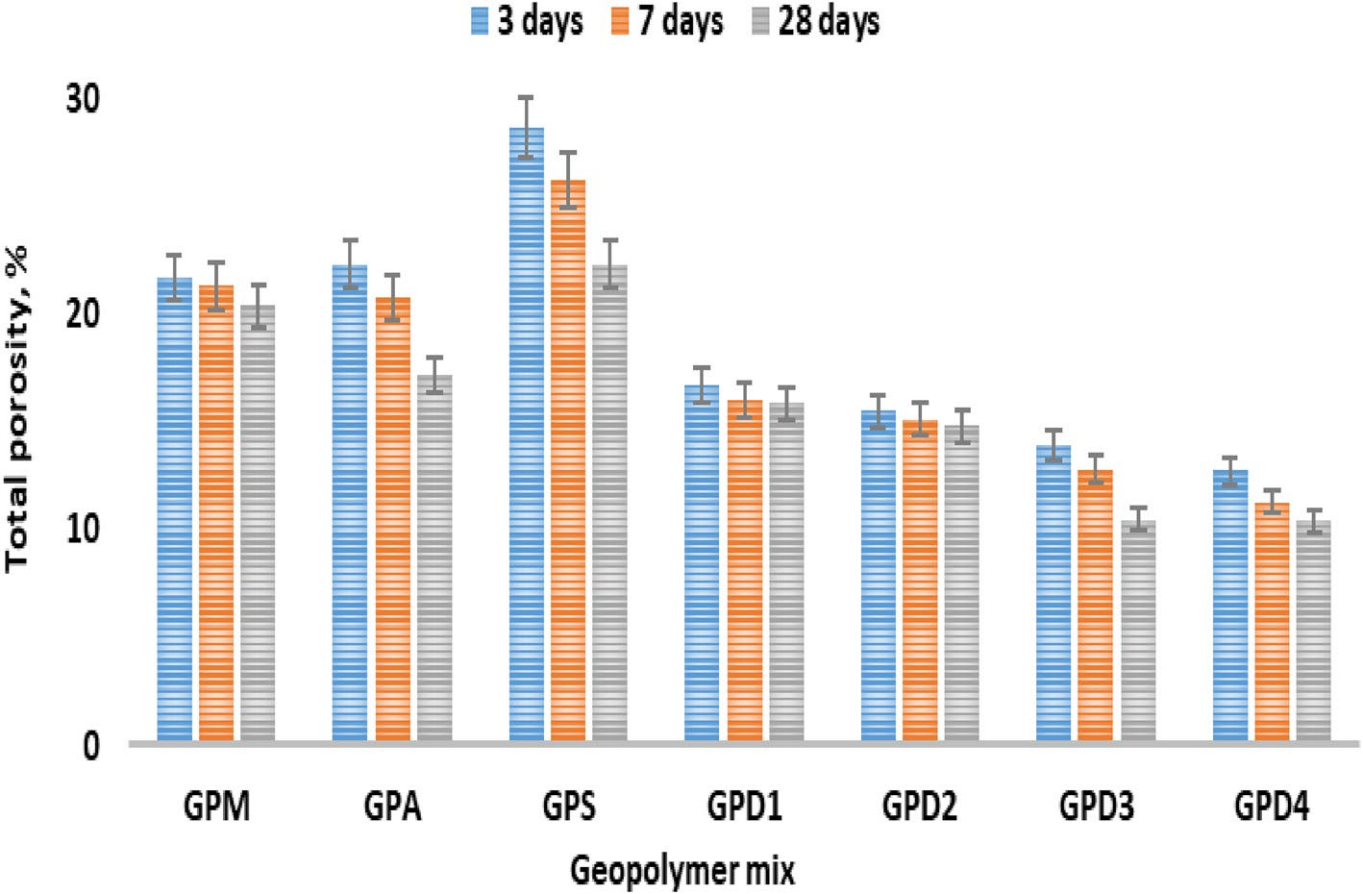
Total porosity of geopolymer variation hydrated for 3, 27 and 28 days.

The TP decreases when the hydration rate increases. The TP of geopolymer pastes report that the low rate of calcium (aluminate & silicate) hydration products generated by the fillers CKD, AP and SF activity are what cause the composite pastes' higher TP outcomes in the geopolymers hybridization, as shown in Fig. 5. TP rises as the open pores of the specimen shrinks, allowing the alkali and lime to combine and produce C–A–H, C–S–H, and C-ASH respectively. TP, increases by SF% and AP% addition, as both GPD and GPM blends shows lower TP value than the GPD1 to GPD2 mixes due to the impact of the CKD crystallinity shape^49,50^. The mixtures with 15.0 wt% CKD exceeds the GPD1 sample (GPM) due to the reductions in the WC/P percent, which enhances the MCS features, the conjugated 15.0–25.0 wt% CKD + 85.0–75.0 wt% MK has the lower TP, value among the others geopolymers. The findings of the four GPD mixes show a reduction in the MCS, and density when the CKD is replaced with 15.0 to 25.0% 85.0–75.0% MK.

#### Water absorption

**Figure 6:**
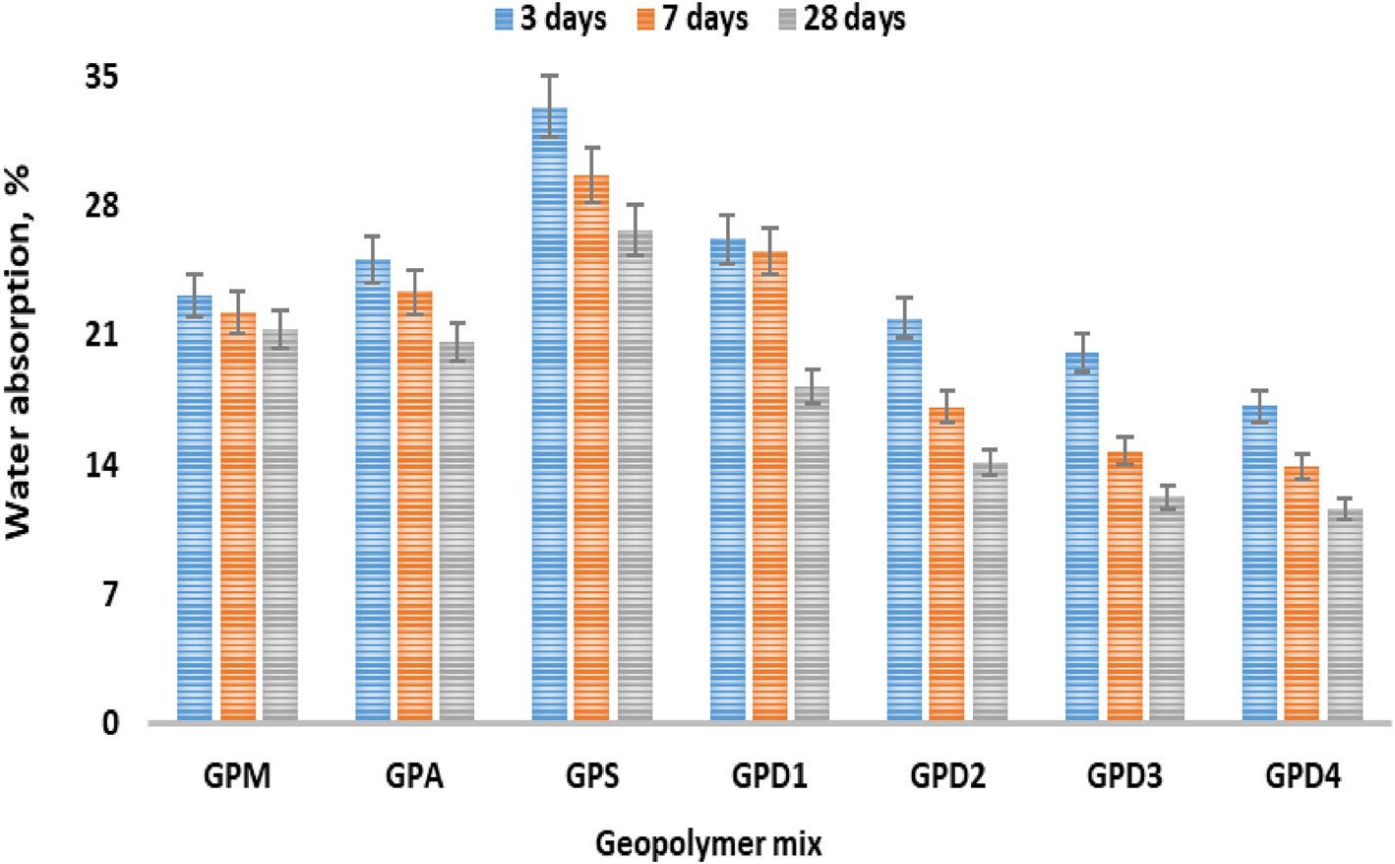
Water absorption of geopolymer variation hydrated for 3, 27 and 28 days.

The results of water absorption, apparent porosity, of geopolymers specimen GPD manufactured with 15–25% CKD, resulted in lesser values of water absorption, apparent porosity and sorptivity when compared to the ones produced with SF% and AP% as shown in Fig. 6. GPS geopolymer paste shows the highest water adsorption rate, which may have attributed to low solubility and high impurities content in the sample. Eventually, the percentage of water absorption decreased with the increase in the hydration age as a result of the progress of the geopolymer formation reaction whose products are deposited inside the open pores in the geopolymer paste^51^. However, the water absorption rate increases with the addition of alumina and silica fume^52,53^, then decreases with the addition of MK, and continues to decrease as the metakaolin ratio is replaced by CKD as active filler and may be attributed to the fact that higher alkali content in the mix gives better reactivity with the cement kiln ash resulting in denser microstructure and less water adsorption^54^. Deep investigation, of water adsorption variation of geopolymer hydrated for 3, 27 and 28 days show that GPD4 paste recorded 18.79% water absorption and 1.61% apparent porosity, whereas geopolymer paste of GPS and GPA showed comparatively lower corresponding values of 35.42% and 27.54% respectively. The geopolymers pastes have the follows order regarding their water adsorption results, e.g.; GPM > GPD4 > GPD3 > GPD2 > GPD1 > GPS > GPA. Eventually, GPD pastes shows better BD and lower TP, and as CKD%, content increase water adsorption increases due to increases in alkali content.

### Characterization of the developed geopolymer

#### Morphology and microstructure

**Figure 7:**
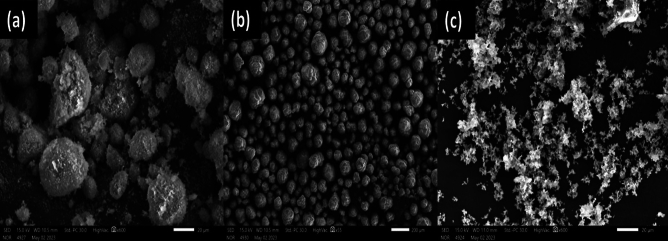
SEM morphology for silica fume (**a**), alumina (**b**) and CKD (**c**).

The SEM morphology for the precursors has been carried out. Figure 7a, report the morphology for silica fume (SF) microstructure, and prove the powder is ultra-fine and amorphous shape. Alumina powder (AP), morphology shown in Fig. 7b, shows that the microstructure is well crystalline and well arranged in amorphous Skelton particles. The CKD morphology, as shown in Fig. 7c, confirms that the microstructure is heterogeneously dense with asymmetrical particles in shape.

**Figure 8:**
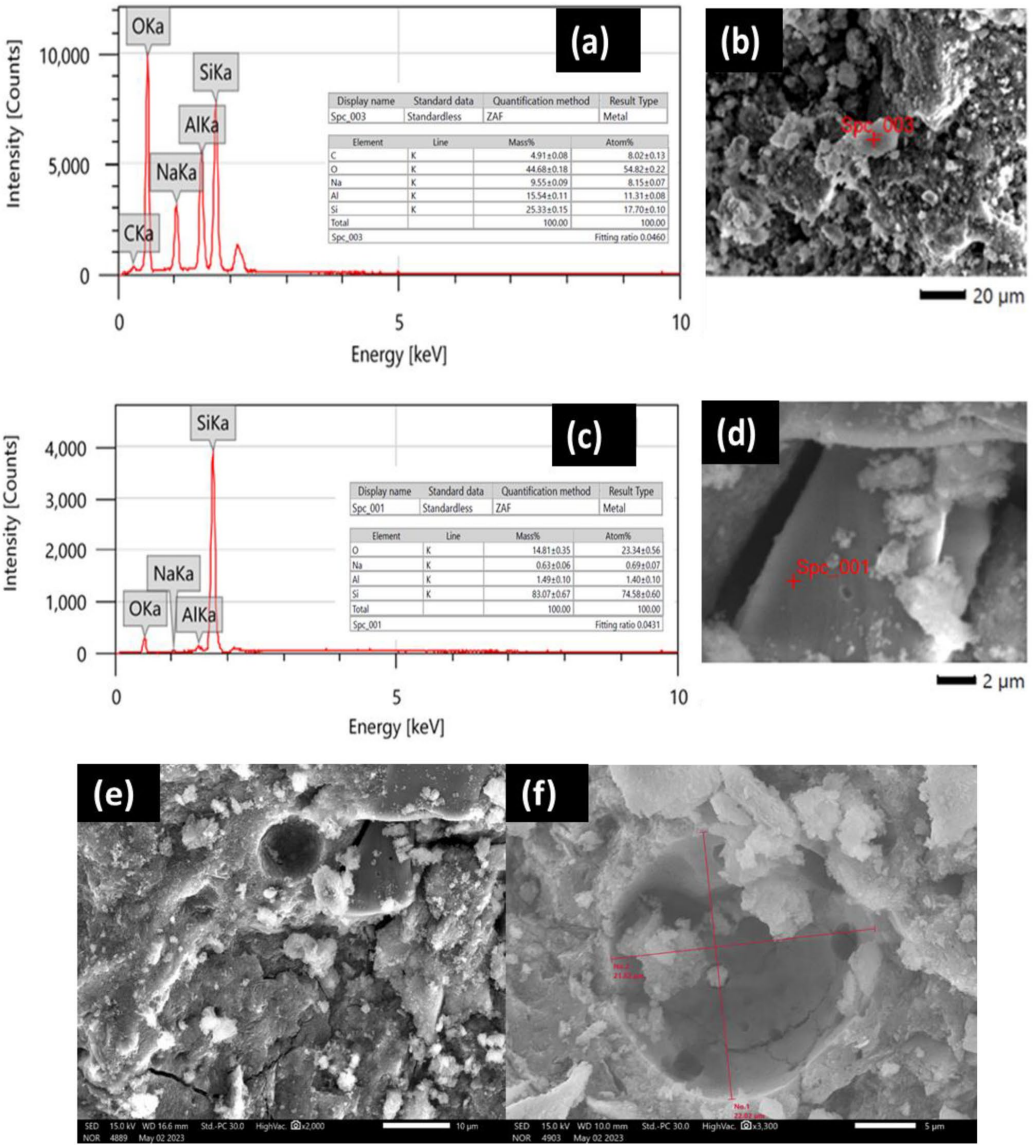
EDX (**a**) and SEM morphology of (**b**) unreacted metakaolin residue appears as hexagonal sheets, EDX (**c**) and SEM morphology (**d**) of quartz residue appears as a smooth surface and SEM images for the pores are few in number, small in size and slits are uninterrupted (**e**), and the pores larger than 10 µm in diameter of geopolymer samples prepared from metakaolin filled with cement kiln dust (**f**).

It was discovered from the above results that the sample of geopolymer prepared from metakaolin alone, GPM, was the best in mechanical properties, followed by the samples of geopolymer prepared from metakaolin replaced with cement kiln dust, GPD1-4, while the sample of geopolymer prepared from metakaolin replaced with alumina GPA, as well as the sample of geopolymer prepared from metakaolin replaced with silica GPS, was the weakest in mechanical properties. However, the SEM images confirm the previous conclusions. Indeed, the fine structure of the geopolymer sample prepared from metakaolin alone, GPM, appeared to be coherent, as shown in Fig. 8a,b, due to the presence of an identical composition of the geopolymer, which appears as white granules or flakes, in which the residues that did not interact are embedded, such as the identical structure of metakaolin, which appears as hexagonal sheets. As in Fig. 8c,d, and quartz which appears as smooth surfaces in appearance. Even the pores in the fine structure of the geopolymer sample are few in number and small in size, as it appears from the scale that their diameter does not exceed 10 µm, and the cracks are not intertwined and narrow, as in Fig. 8e,f.

**Figure 9:**
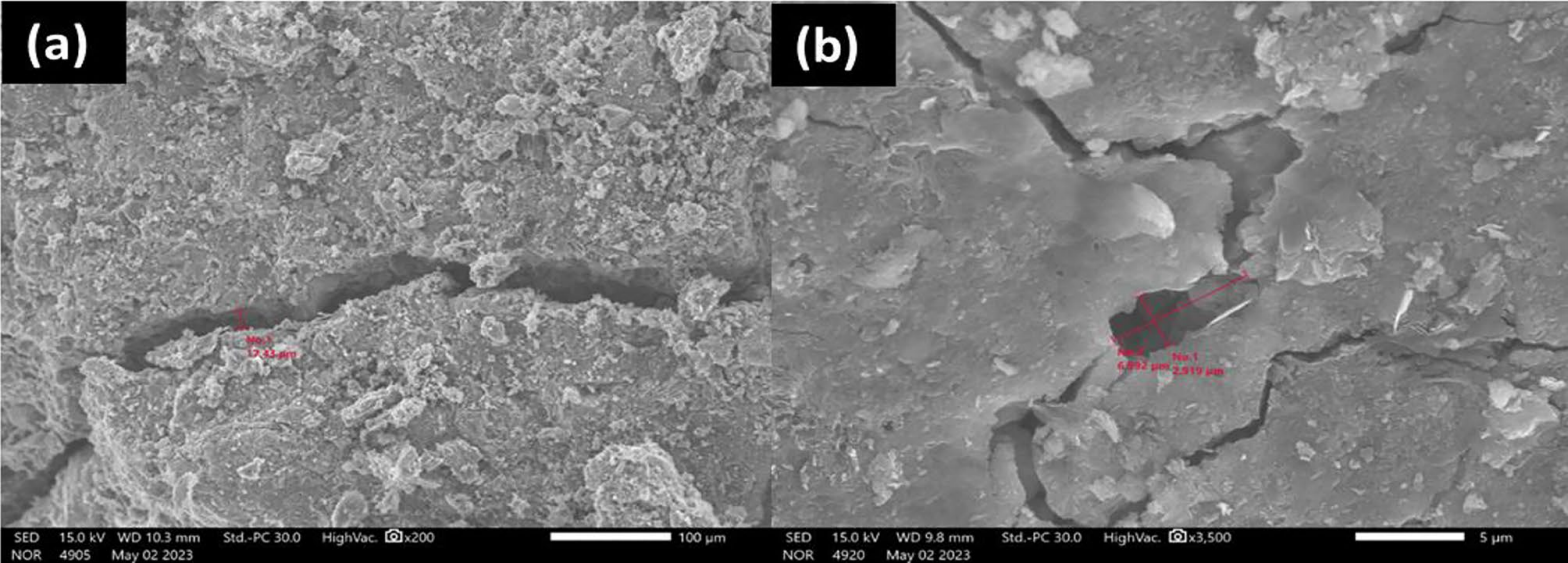
SEM image of wide cracks in geopolymer samples prepared from metakaolin replaced with cement kiln dust (**a**) and entanglement of cracks increases with the percentage of cement kiln dust (**b**).

Whereas, it appeared in the geopolymer samples prepared from metakaolin replaced with cement kiln dust GPD1-4, the presence of many pores, as the scale appears to be more than 10 µm in diameter, as in Fig. 9a, as well as wider cracks. The intertwining of cracks and pores increases as the percentage of metakaolin replaced by CKD, increased as shown in Fig. 9b. Therefore, the mechanical properties decreased as the percentage of cement kiln dust increased. It was also found that there is an identical composition of calcium-rich geopolymer as a result of the presence of cement kiln dust rich in calcium^55^.

**Figure 10:**
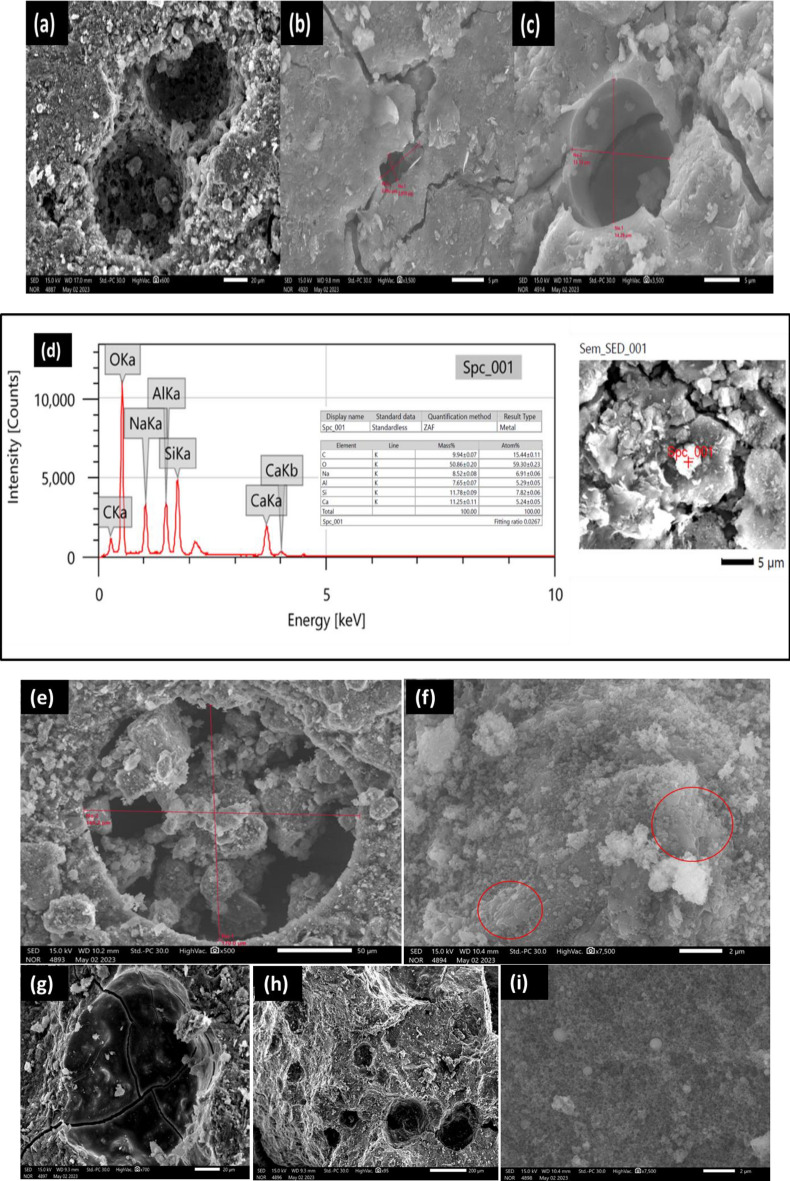
SEM images for the entanglement of pores increases with excess of cement kiln dust (**a** and **b**), Identical composition of calcium-rich geopolymer in geopolymer samples prepared from metakaolin replaced with cement kiln dust (**c**), the EDX of calcium-rich geopolymer in geopolymer samples prepared from metakaolin replaced with cement kiln dust (**d**), SEM images show large pores with a diameter of more than 200 μm in the geopolymer sample prepared from alumina-substituted metakaolin (**e**), many metakaolin residues with phyllosilicates in a geopolymer sample prepared from alumina-substituted metakaolin (**f**), and numerous large pores in the geopolymer sample prepared from metakaolin replaced with silica earth (**g**, **h**), and the silica fume balls embedded in the microstructure in a geopolymer sample prepared from metakaolin replaced with silica dust (**i**).

The intertwining of cracks and pores increases as the percentage of metakaolin replaced by cement kiln dust increased as shown in Fig. 10a,b. Therefore, the mechanical properties decreased as the percentage of cement kiln dust increased. It was also found that there is an identical composition of calcium-rich geopolymer as a result of the presence of cement kiln dust rich in calcium, as shown in Fig. 10c. As for the fine structure of the geopolymer sample prepared from metakaolin replaced by alumina GPA, it contains many large pores, as it appears from the scale that its diameter exceeds 200 µm, as shown in Fig. 10d. It also contains a large amount of metakaolin residue, which appears in the form of multilayered lamellar structures, as phyllosilicates. Figure 10e. Also, the geopolymer sample prepared from metakaolin replaced with silica dust GPS contains many cracks penetrating the pores as in Fig. 10f, and the largest percentage of large pores appear in it as in Fig. 10g,h, in addition to containing silica fume, which appears as balls embedded in the fine structure of a sample Geopolymer, as shown in Fig. 10i, are the weakest in mechanical properties.

#### FT-IR analysis

**Figure 11:**
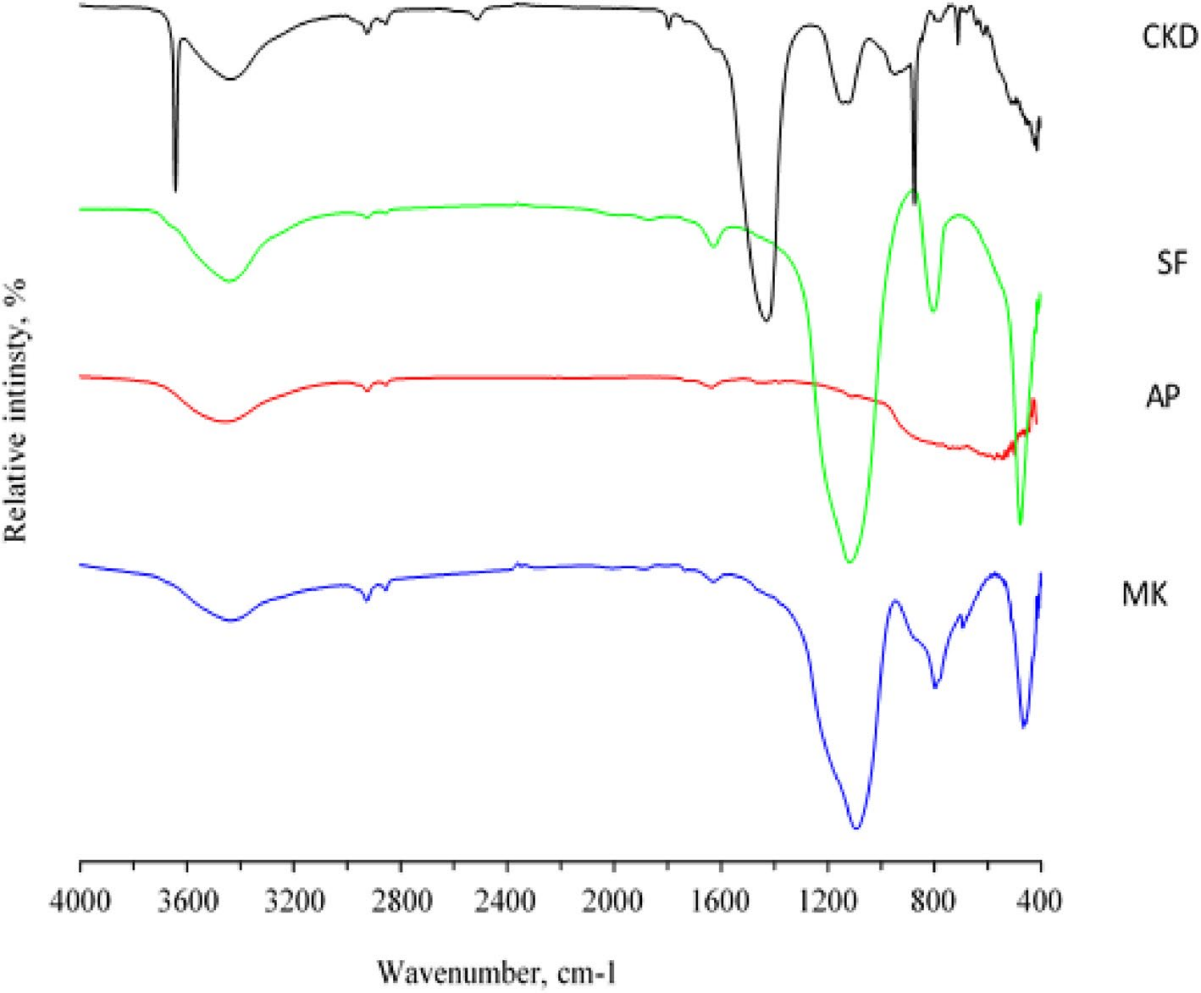
FTIR spectra of Metakaolin (MK), Alumina Powder (AP), and Silica Fume (SF).

Fourier transform infrared spectroscopy (FTIR) is an analysis technique used to provide information about vibrational shifts and the stiffness of chemical bonds within and between molecules. Figure 11, shows the FT-IR spectra for the raw materials used. The spectrum of metakaolin (MK) is characterized by the presence of a double band of O–H between 3550 and 3700 cm^−1^. Therefore, it appears very weak as a result of burning kaolin and converting it to metakaolin, which explains the scarcity of chemical water in the structure of kaolin. After calcination at 800 °C these bands disappear, confirming the absorbed beaks from 1150 cm^−1^^56^. Another range of metakaolin from 2800 to 3600 cm^−1^ also refers to the O–H bond of low energy due to the moisture and adsorbed water disappearing from the geopolymer. At about 1630 cm^−1^ there is a band of aqueous functional group (H–O–H) in CKD. The range between 1100 and 1330 cm^−1^ in the alumina powder (AP), product is attributed to the resulting O–C–O functional group. Finally, silica fume (SF), shows a broad band at 1170 cm^−1^, of O–H, where the band appears at 3500 cm^−1^ is for free silica^57^. In the FTIR spectrum of MK, OH stretch vibration is recorded at 3695 cm^−1^, 3620 cm^−1^, and 3445 cm^−1^, H–O-H bend appear at 1650 cm^−1^, Si–O stretch at 1030 cm^−1^, and Al–O–Si stretch at 910 cm^−1^. For AP, there is OH-stretch at 3690 cm^−1^, 3625 cm^−1^, and 3450 cm^−1^, H–O-H bend at 1635 cm^−1^, Si–O stretch at 1035 cm^−1^, and Al–O–Si stretch at 915 cm^−1^. Concerning SF, the OH-stretch appeared at 3685 cm^−1^, 3620 cm^−1^, and 3440 cm^−1^, H–O–H bending and Si–O stretch were recorded at 1630 cm^−1^, and 1030 cm^−1^, respectively. The FTIR spectra of MK, AP, and SF exhibit similarities, indicating the presence of siloxane linkages and hydroxyl groups in the three compounds. The presence of other functional groups in addition to variations in the quantity and arrangement of hydroxyl groups could be the cause of the variations in peak intensities and positions. Si–O–Si bonds are formed and stabilized in part by hydroxyl groups, which also start the geopolymerization process. Silicate and aluminate species can be connected by hydroxyl groups, which function as bridge ligands and aid in the creation of Si–O–Al–O chains. Furthermore, hydroxyl groups have the ability to coordinate with silicon atoms, strengthening the geopolymeric network's overall stability and reinforcing the Si–O–Si connections.

The following instances highlight the significance of the distinctions between physical and chemical properties:

- Physical characteristics: The infrared spectrum of metakaolin (MK) has high peak intensity, suggesting the presence of many hydroxyl groups. Metakaolin (MK) exhibits enhanced thermal and electrical conductivity as a result.

- Chemical characteristics: Compared to alumina powder (AP) and silica fume (SF), metakaolin (MK) exhibits distinct peak locations in the infrared spectrum. This causes metakaolin (MK) to behave differently chemically and reactivity-wise than silica fume (SF) and alumina powder (AP)^1,5^

**Figure 12:**
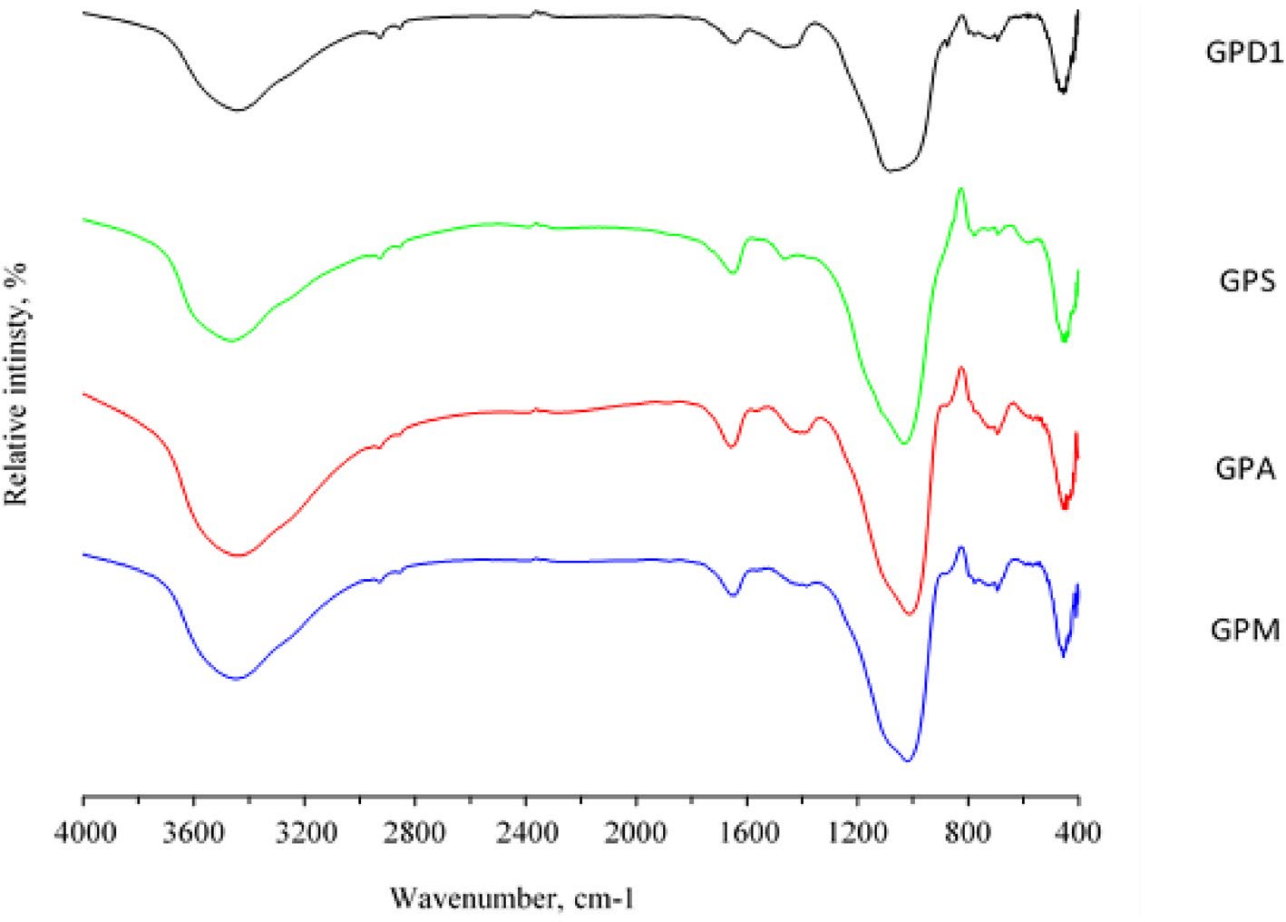
FTIR spectra of metakaolin based geopolymer (GPM), as well as metakaolin/alumina (GPA), metakaolin/silica fume (GPS), and metakaolin/ cement kiln dust (GPD1) based geopolymers.

Different types of FTIR spectra for geopolymers solidified at 28 days are shown in the Fig. 12. The different bands that appear in the spectra are described as follows: Geopolymer metakaolin, alumina, silica fume and cement kiln dust (GPM, GPA, GPS and GPD). The figure shows different types of FTIR spectra for the geopolymer after 28 days in addition to the raw materials used. The spectrum of kaolin is characterized by the presence of a double band of O–H between 3550 and 3700 cm^−1^. Here it appears very weak as a result of burning kaolin and converting it to metakaolin, which explains the scarcity of chemical water in the structure of kaolin. After calcination at 800 °C these bands disappear, confirming the shape^58^. Another range of metakaolin from 2800 to 3600 cm^−1^ also refers to the O–H bond of low energy due to moisture and adsorbed water disappearing from the geopolymer. At about 1630 cm^−1^ there is a band of aqueous functional group (H–O–H) in kaolin, metakaolin, and geopolymer. The range between 1300 and 1430 cm^−1^ in the geopolymer product is attributed to the resulting O–C–O functional group from carbonation that produces calicienite (KHCO_3_) which has already been observed in X-ray diffraction plots. It has also been shown that the intensity of the bands characterizing the functional groups Si–O–Si (550 cm^−1^) and Si–O–Al (620 cm^−1^) decreases when kaolin is calcined, which explains the destruction of the Si–O and Al–O bonds of mud. The range from 900 to 1100 cm^−1^ is quite large. In all the different types of geopolymers under study, the FTIR spectra are similar, but in the case of the type composed of a mixture of metakaolin with alkaline activator sodium metasilicate, the aluminosilicate bond will be sharper and denser, which leads to an increase in its mechanical strength. While in the rest of the types, it was noticed specifically at 445 cm^−1^ that the Si–O bonds increase within the basic aluminum silicatetetrahedron. The porosity increases very much in the case of silicate, which weakens the toughness. Also, the increase of the Si–O bonds within the basic aluminum silicate in the case of the type containing alumina affects the chemical composition and then the tetrahedral spatial shape, so the bonds weaken, so their cohesion decreases and their hardness decreases, as is the case also in the case of Cement kiln dust.

**Figure 13:**
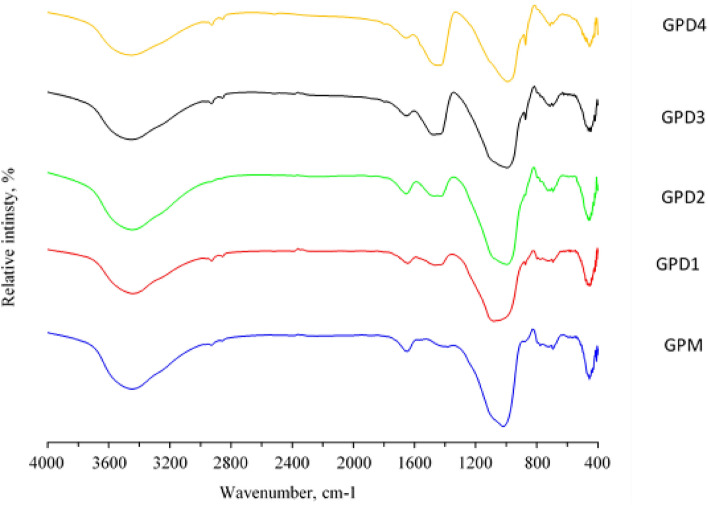
FTIR spectra of metakaolin based geopolymer (GPM), as well as metakaolin/cement kiln dust based geopolymers (GPD1-4).

Figure 13, is also revealed the appearance of an intense and sharp band in the geopolymer spectrum of Si–O–Al at 620 cm^−1^. It increases, of course, in the alumina-containing type, and almost fades in the case of silica fume and increases in the case of cement kiln dust, and this also explains the variation in strength. Hardness and others in different types of geopolymer pastes^59^. The different FTIR spectra of the geopolymer show the content of a mixture of metakaolin and cement kiln dust in mixture weight percentages of 50%, 35%, 25% and 15% after 28 days, completely similar until the range from 1300 to 1430 to 800 cm^−1^ which explains the O–C–O functional group resulting from carbonation^60^ that produces calicinitrate (KHCO_3_) as explained previously. The range between 900 and 1100 cm^−1^. These ranges are also allocated to the symmetrical and asymmetric vibrations of Si–O–Si valence bonds, which explains the emergence of new phases that appear and disappear as the proportions of cement kiln dust increase, which affects its physical and mechanical properties.

#### XRD and phase composition

**Figure 14:**
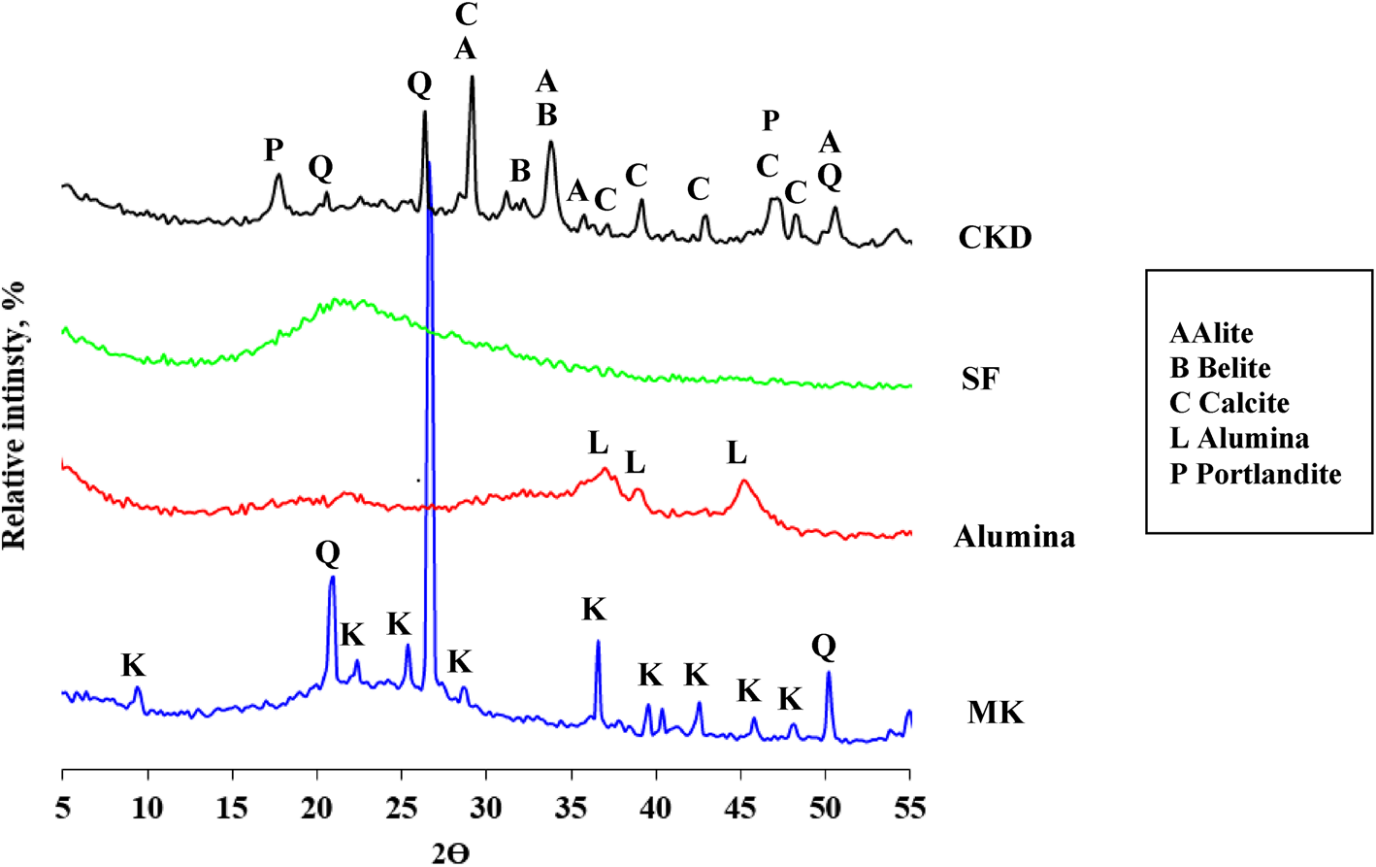
X-ray diffraction patterns of raw materials MK, alumina, SF, and CKD.

**Figure 15:**
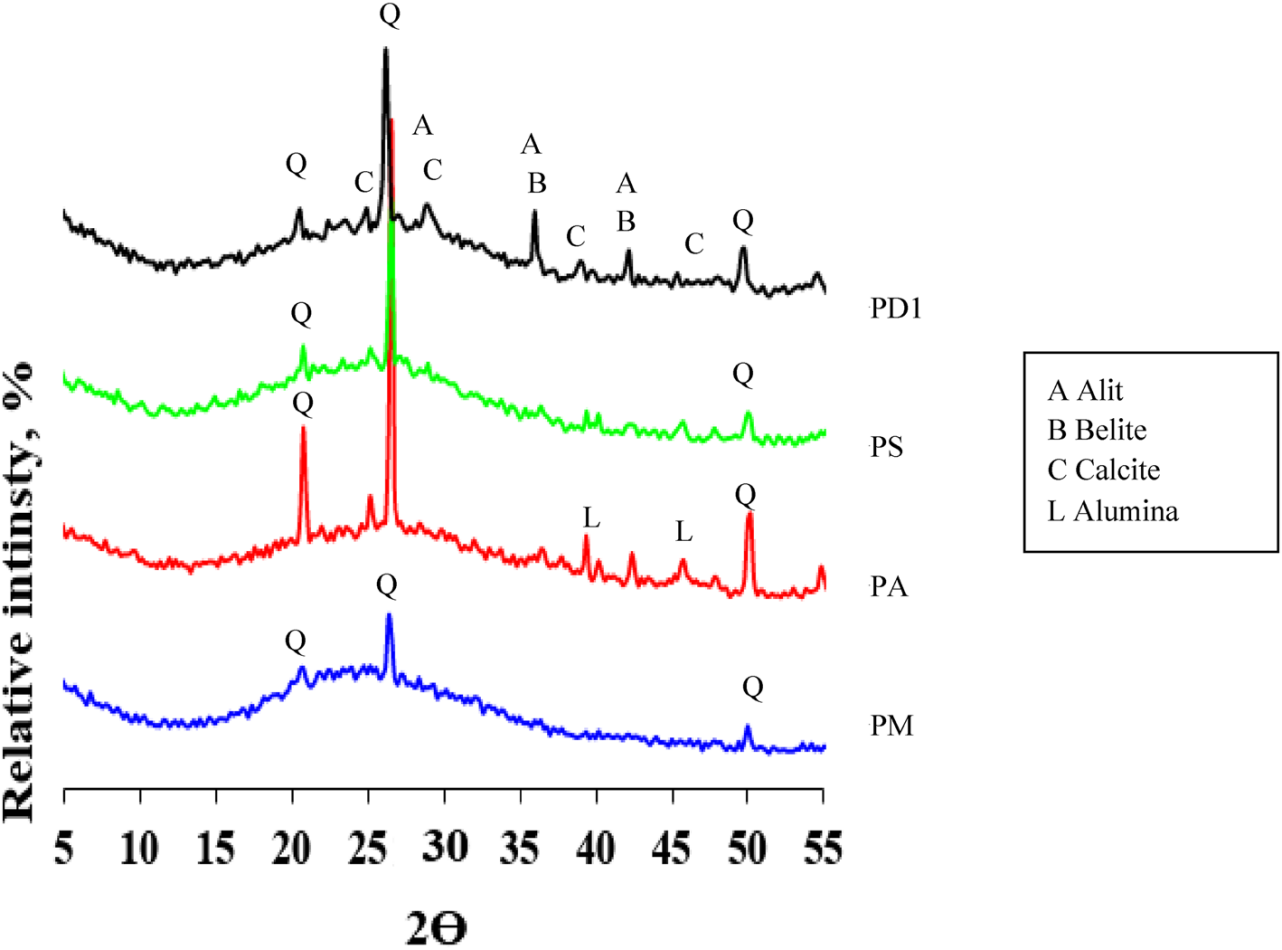
X-ray diffraction patterns of geopolymer pastes GPM, GPA, GPS, and GPD1 hydrated for 28 days.

**Figure 16:**
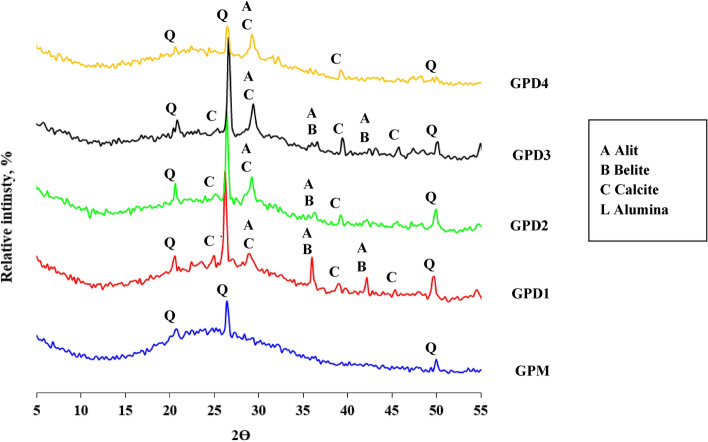
X-ray diffraction patterns of geopolymer pastes GPM and GPD1-GPD4 hydrated for 28 days.

Metakaolin contains amorphous substance resulting from the burning of kaolin with the presence of quartz and remnants of kaolinite crystals whose conversion to metakaolin was incomplete as a result of the insufficient burning conditions. The alumina contains amorphous alumina phase with little crystalline alumina as indicated by the diffraction peaks that appear at the values 35:45 Fig. 14. Silica fume contains an amorphous silica phase, as can be seen in the diffraction pattern, which takes on an amorphous character in the range from 2Ɵ = 15 to 30 (Fig. 15). Cement kiln dust contains clinker minerals, alite and belite, in addition to calcite, quartz, and small percentages of other minerals (Fig. 16)^61^. The treatment of metakaolin with the alkaline activated solution led to the formation of amorphous geopolymer containing the remnants of quartz crystals, while the kaolinite crystals disappeared, indicating the complete dissolution of the kaolinite mineral in the alkali activated solution^62^. Partial substitution of metakaolin by alumina reduced the amount of amorphous geopolymer formed as indicated by the height of the quartz peaks. It is also noted that the alumina crystals did not disappear, indicating the low solubility of the alumina mineral in the activated alkaline solution compared to kaolinite. As well as the appearance of a new phases at values of 2Ɵ = 25.2 and 42.4, which may be from the alumina-rich geopolymer phases^63^. While the partial replacement of kaolin with silica fume resulted in an improvement in the amount of amorphous geopolymer formed compared to the case of alumina, which is indicated by the decrease of the quartz peaks, an indication of the solubility of the amorphous silica mineral in the alkaline activated solution with a higher degree than that of alumina^64,65^. While the partial replacement of metakaolin with silica dust resulted in an improvement in the amount of amorphous geopolymer formed compared to the case of alumina, which is indicated by the decrease of the quartz peaks, an indication of the solubility of the amorphous silica mineral in the alkaline activated solution with a higher degree than that of alumina. Partially replacing metakaolin with cement kiln dust is the best compared to the previous additives, alumina and silica dust, as evidenced by the improvement in the amount of amorphous geopolymer formed. As evidenced by the continued decrease of the quartz peaks, an indication of the rapid interaction of cement kiln dust minerals with the alkali activated solution. Especially the Portlandite mineral, which has completely disappeared, which indicates its dissolution and its participation in the formation of calcium-rich geopolymer phases. This was observed from the decrease in the setting time of geopolymer slurries to which cement kiln dust was added^66–69^.

## Conclusion

In this research article, we would like to present the possibility of using agro-wastes and cement kiln dust products for the synthesis of geopolymers. Scientific articles focusing on the use of by-product wastes to produce geopolymer cement. Examples of upcycling industrial construction, demolition, and cement waste containing alumina and silica, which are toxic to the environment, into functional geopolymer materials will be discussed. Additionally, the paper focuses on innovative applications and physicochemical properties of functional geopolymer materials. The main findings of this study are summarized below:

1. The geopolymers are a promising alternative to OPC cement owning to their ability to incorporate industrial by-products from other industries such as alumina powder, silica fume, and cement kin dust.
2. The physico-mechanical features of the metakaolin geopolymer composite were improved by the addition of CKD; this attributed to excess CaO which acts as active dynamic filler, improving the microstructure of the blends. In addition to the free alkali content which play an important role during hydration.
3. Although SF possesses free silica, it has poor effect on the hydration process.
4. The compressive strength of geopolymers is directly proportional to the ratio of the alkali liquid.
5. The optimum mixes were GPD1 (15% of CKD + 85% MK and Alumina solution (55 wt%)) and GPD2 (25% of CKD + 75% MK + Alumina solution (55 wt%)).

Eventually, geopolymer is considered green mainly due to the reduction of CO_2_ emissions, the reduction of energy used during the production process and the use of raw materials treated as "waste" in construction for their production. All these features are focused on reducing global warming and reducing the amount of waste produced by the formulation of these green geopolymer materials. However, it is worth paying attention to the study on carbon footprint detection for geopolymer production due to conflicting literary values.

## Data Availability

The datasets used and/or analyzed during the current study available from the corresponding author on reasonable request.
